# Why uterine transplantation requires us to rethink the role of the pre-conception welfare principle

**DOI:** 10.1093/jlb/lsac028

**Published:** 2022-10-11

**Authors:** Laura O’Donovan

**Affiliations:** Department of Politics, Philosophy and Religion, Lancaster University, Lancaster, UK

**Keywords:** uterine transplantation, assisted reproduction, uterus, gestation, pre-conception welfare, reproductive autonomy

## Abstract

Uterine transplantation (UTx) is a programme of treatment aimed at providing a unique solution to absolute uterine factor infertility, enabling patients to have children as a result of their own pregnancies. As a transplant procedure performed for fertility purposes it may be thought obvious that the welfare of any children created should be assessed prior to treatment provision. However, major concerns about the breadth and scope of such requirements, and the potential threat they pose to patients' reproductive autonomy, have been raised. In this paper, I analyse novel questions regarding the role of the pre-conception welfare principle in UTx. After outlining traditional critiques of the principle, I focus on the unique issues raised by its application in the two areas of medicine occupied by UTx. As a treatment for a particular form of infertility, I explore whether law and policy regulating traditional assisted reproductive technologies applies equally to the case of UTx, and whether a distinction (in welfare terms) does and should exist between fertility treatment involving gametes and embryos and gynaecological surgery for fertility purposes. As a quality-of-life-enhancing transplant, I consider and reject proposals in favour of using pre-conception welfare considerations to inform patient listing and the allocation of deceased donor uteri on the grounds that such assessments may both compromise patient autonomy and lead to unjust discrimination against particular patients or groups of patients.

## I. INTRODUCTION

The field of reproductive medicine has experienced significant advancement and expansion in recent years, and treatments that were once thought to be distant possibilities are now a firm reality. Uterine transplantation (UTx) is one such treatment that has undergone significant evolution. At the cross-section of two distinct areas of medicine, reproductive medicine and transplantation medicine, it aims to provide those diagnosed with absolute uterine factor infertility (AUFI)—that is, complete infertility due to the absence, or lack of function, of the uterus^1^—with a third option for family building. For those with AUFI, traditional forms of assisted conception cannot facilitate pregnancy, and other existing options for family building (surrogacy or adoption, which each has its own legal, ethical, financial, religious, etc. complications) are not able to offer the unique experience provided by UTx.^2^

As we begin to see the clinical translation of UTx in a number of centers^3^ and as clinical trials continue around the world,^4^ it is crucial not only that any remaining medical and surgical questions are answered but also that relevant ethical and legal issues are carefully examined. In the context of assisted reproductive technology (ART), many countries either mandate or advise that fertility clinics assess the welfare of any not-yet-conceived child prior to offering treatment.^5^ As a hybrid transplant-reproductive treatment, patients seeking UTx will, thus, also be subject to child welfare assessments in different centers around the world as in vitro fertilization (IVF) is a necessary part of the treatment program. Beyond emphasizing welfare considerations as a threshold tool to determine treatment access, some authors have suggested that such assessments could be used to assist with the allocation of deceased and living non-directed donor uteri.^6^ While there exists a rich academic literature discussing the ethical and legal permissibility of the pre-conception welfare principle (PCWP) in relation to more familiar forms of assisted reproduction, including pre-implantation genetic testing (PGT-M/SR), to date there has been limited consideration of the presence and scope of child welfare assessments in the novel context of UTx. This paper seeks to fill this gap asking what role child welfare considerations will and should have in our ethical and legal reasoning about UTx, focusing in particular on the legal framework in the UK.

Given the rapid pace with which this form of ART has developed, it is imperative that we move from considering whether UTx is possible to considering how this advance will be implemented and governed in practice, particularly given its dual nature as both a transplant and reproductive treatment. This raises four important questions of legal and ethical significance regarding: first, whether assessments of a hypothetical child’s welfare are justified in the context of assisted reproduction generally; second, whether compulsory assessments of a hypothetical child’s welfare are justified in the context of UTx in particular; third, whether there is a role for pre-conception welfare considerations in the allocation of deceased donor uteri, and if so, whether this should be a threshold test only, or, aimed at optimization/maximization; and finally, what the form and consent of such child welfare assessments should be.

The structure of this article is as follows. Part II summarizes the current clinical limitations of UTx, providing vital scientific and legal background about the hybrid nature of UTx as both a transplant and assisted reproductive treatment. In Part III, the rationale for child welfare assessments in assisted reproduction is considered, focusing in particular on the operation of the welfare principle in the UK. It explores the reasoning that is normally used to justify and ground the welfare principle as well as criticisms leveled against it, and examines what and whose interests the principle is designed to protect. Part IV continues to examine whether the current rationale for assessing child welfare applies equally to the case of UTx and explores whether a distinction (in welfare terms) should exist between fertility treatment involving gametes and embryos and gynecological surgery. Part V moves on to consider the role of welfare reasoning in the allocation of donor uteri. It examines and rejects proposals for the use of welfare as a threshold criterion to determine access to transplant waiting lists, and further, cautions against allowing welfare considerations to inform prioritization frameworks and be utilized as a tool to optimize the allocation of deceased donor uteri.

## II. THE LIMITS OF UTERUS TRANSPLANTATION AS A TREATMENT FOR INFERTILITY

### II.A. Natural Pregnancy as a Medical (Im)possibility?

In order to explore the proper place of the PCWP in UTx, it is first important to set out why UTx involves two different areas of medicine. The treatment (comprised of the creation of embryos through IVF; retrieval and transplantation of a donor uterine graft into the recipient; immunosuppressive therapy; the transfer of embryos to the donated womb; and cesarean section) enables the recipient to carry their own child to term and experience abdominal birth.^7^ While it may enable gestation, UTx cannot currently facilitate natural conception through sexual intercourse as the uterine graft is not surgically connected to the patient’s fallopian tubes,^8^ and the donor oviducts are not transplanted as part of the procedure.

It should be noted that the presentation of AUFI differs between individuals and according to the underlying cause. For example, the cause of AUFI may be congenital, resulting from conditions such as Mayer-Rokitansky-Kuster-Hauser (MRKH) syndrome, or acquired as a result of, for example, gynecological cancer necessitating surgical removal of the uterus; or conditions affecting the uterine functionality.^9^ Patients with MRKH typically present with congenital absence or underdevelopment of the upper vagina and uterus but retain normally functioning ovaries and the presence of fallopian tubes (though this may not always be the case).^10^ What this means is that for those who want to reproduce and who retain ovarian-tubal function, theoretically all that is lacking is the uterine graft,^11^ which, if connected to the fallopian tubes, may potentially facilitate spontaneous and natural conception leading to established pregnancy. For those with acquired AUFI on the other hand, and depending on the cause, total hysterectomy with unilateral or bilateral salpingo-oophorectomy (removal of one of both ovaries and oviducts) may be required. As a result, transplantation of not only the uterus, but also the oviduct and potentially the ovary would be required to facilitate spontaneous conception.

However, such procedures are not currently medically possible due to the difficulties associated with securing adequate perfusion of the complex vasculature to ensure tubal viability alongside what is already a technically complex and difficult operation to secure viability of the uterine graft. Teams conducting UTx trials are also skeptical about the formation of ovarian-tubal function between a transplanted oviduct and native ovary.^12^ For example, according to Brännström and colleagues, writing in 2010, the chance for natural conception following UTx including the oviduct would be minimal with high risk of ectopic pregnancy.^13^ Added to this, the authors also suggest that the presence of low-grade inflammation in a transplanted oviduct (a typical inflammatory response in transplanted tissue) would likely negatively influence embryo implantation following IVF (assuming ART would be pursued if spontaneous conception did not occur). ^14^

Given previous failures in human transplantation of the oviducts^15^ and only limited success in animal models, current research has thus focused exclusively on techniques for transplantation of the uterine graft absent the fallopian tubes. As natural conception is therefore not physically possible, assisted reproduction consequently forms an integral part of UTx treatment—surgery provides the gestational environment to host a pregnancy, but IVF is necessary to facilitate the establishment of that pregnancy within the uterine environment.

### II.B. The Legal Regulation of Uterine Transplantation in the UK

Having established that, at present, the success of UTx is dependent on IVF (success being understood as pregnancy in the recipient and the live birth of a child), UTx must not be viewed in isolation as a transplant procedure, but as a *program* of treatment involving both transplant and reproductive medicine (UTx-IVF). Acknowledging this, it is clear that in the UK (and in other countries with similar regulatory frameworks governing the provision of assisted conception services), UTx-IVF falls under the remit of two regulatory regimes and two regulatory bodies: (i) legislation governing organ donation and transplantation activities overseen by the Human Tissue Authority, and (ii) legislation regulating human reproductive technologies and embryo research overseen by the Human Fertilisation and Embryology Authority (HFEA).

First, as an organ transplant, UTx is governed by the provisions of the Human Tissue Act (HTA) 2004. The uterus is also defined as an ‘excepted material’ in the regulations accompanying the Organ Donation (Deemed Consent) Act 2019, meaning it falls outside the scope of the new opt-out system, and express consent to donation is required.^16^ Second, UTx is captured by the regulatory framework contained within the Human Fertilisation and Embryology Act (HFE Act) 1990 (as amended). This is because IVF (a necessary component of UTx treatment) is a regulated activity falling under Paragraph 1 of Schedule 2 to the Act, to which the license conditions (including the pre-conception welfare assessment)^17^ contained in sections 12–14A, apply.

Though UTx is governed by two different and unconnected systems of regulation, and overseen by two different and unconnected regulators, this system of dual regulation can accommodate the effective provision of UTx treatment as it is *currently* envisaged.^18^ That, however, is not to suggest that potential policy issues could not arise in this context. For example, Alghrani has warned that guidance is needed to ensure that patients do not incur the expense and physical and/or psychological hardship of IVF treatment in the hope of pursuing UTx, to later find that they are ineligible for UTx treatment.^19^

Greater legal obstacles arise when considering possible future applications of UTx. This is largely due to the constraints of, and gaps in, current regulation, as opposed to the fact of dual regulation. For example, a potentially significant regulatory gap, explored in more detail in section IV, will emerge if the science of UTx evolves to facilitate connection of the uterine graft to the patient’s own or transplanted oviducts making natural conception possible. In this case, UTx would no longer fall within the dual regulatory regime described above and would solely be governed by donation and transplantation laws. This is because the HFE Act 1990 (as amended) applies to activities involving gametes and embryos.^20^ In other words, the license conditions, including the welfare of the child assessment required by s 13(5), would *not* apply to UTx unless it was added as a specified activity requiring a license under Paragraph 1 of Schedule 2 to the Act. Whether this is ethically justifiable is unclear and, as will be shown, indicates more wholesale re-thinking about the regulation of fertility-related treatments generally is required.

## III. THE RATIONALE FOR CHILD WELFARE ASSESSMENTS IN ASSISTED REPRODUCTION

### III.A. The Welfare Principle

The enactment of the HFE Act 1990 was a key development in fertility regulation. The Act not only provided a comprehensive framework for the governance and delivery of fertility treatment and embryo research, but also instituted the regulatory body responsible for the licensing and inspection of fertility clinics and the recording of data on all fertility treatments carried out since August 1, 1991—the HFEA.^21^ S 13 of the Act provides the conditions of licenses for treatment that clinics must adhere to. Paragraph 1 of Schedule 2 to the Act lists a number of activities that may be authorized by a license. They include the creation, procuring, keeping, testing, processing, and distribution of embryos and gametes.^22^ The relevant license conditions thus apply to all cases of assisted fertility treatment involving embryos and gametes (IVF with or without intracytoplasmic sperm injection (ICSI) or PGT-M/SR and intrauterine insemination). Almost 20 years since its passing, the 1990 Act was amended by the Human Fertilisation and Embryology Act 2008 (hereafter the 2008 Act). S 13(5)—the PCWP—in its amended form provides that:

[a] woman shall not be provided with treatment services unless account has been taken of the welfare of any child who may be born as a result of the treatment (including the need of that child for supportive parenting), and of any other child who may be affected by the birth.^23^

### III.B. What and Whose Interests? The Justification for Assessing Child Welfare

Noting that the purpose of current research into UTx is the treatment of AUFI, that is, it is explicitly and solely being pursued to enable patients diagnosed with AUFI to gestate and give birth to their own child, it is necessary to explain the purpose of child welfare assessments in the fertility context, and identify any generalizable shortcomings inherent in the process. The welfare principle, as it features in fertility regulation and policy in the UK, applies to both existing and future hypothetical children.

The origins of this principle in the UK are to be found in the long history of the law’s focus on the paramountcy of child welfare in disputes regarding child custody, upbringing, and administration of the child’s property.^24^ To those debating the passing of the original statutory framework governing fertility and embryo research, the expansion of a child welfare principle into this area of law seemed an obvious compromise and the welfare clause was added as an amendment to ensure the Bill passed into law after another amendment that would have confined IVF to married couples was narrowly defeated.^25^ Indeed, as Jackson has noted, its inclusion was neither questioned nor defended.^26^ Such reasoning appears to be based on the fact that the purpose of fertility treatment is the creation of children—their interests, fundamental as they are in child law, should thus also be considered. Indeed, frequent references to the paramountcy of child welfare in the law about children appear throughout the debates in both Houses of Parliament on the passing of the Human Fertilisation and Embryology Bill, and the inclusion of a principle considering the welfare of a to-be conceived child was overwhelmingly supported.^27^ This support, however, was not accompanied by detailed legal, philosophical, and sociological reasoning, meaning that the justification for the inclusion of a welfare principle in this area is mainly derived from convention.

A further possible justification for the inclusion of child welfare assessments in fertility treatment relates to an argument about the effective use of resources. This is relevant in the case of publicly funded fertility treatment, such as that provided by the NHS, where pressure on resources is significant. Presumably, if the aim is to allocate resources effectively, this would require clinicians to provide fertility treatment only to those patients who will make the best parents. This implies three things: (i) determining the circumstances within which child welfare will be maximized—the best parents being those who can provide the best life for a future child; (ii) selecting prospective parents according to their ability to meet this standard; and (iii) determining the point below which offering treatment would be insufficiently cost-effective. The welfare principle contained in s 13(5) however does not go this far and indeed, in practice, neither the HFEA nor treating clinicians have interpreted the requirement as such.

Since 2005, the HFEA has guided clinics to base their approach on a presumption to treat.^28^ This presumption was formally introduced in the HFEA’s 8^th^ Code of Practice (CoP)^29^ and remains an important guiding principle in the most recent Code of Practice, published in January 2019.^30^ Furthermore, in various iterations of its guidance documents since 2005, the HFEA has framed the discharge of clinics’ statutory responsibility to consider child welfare as a risk assessment. To this end, its CoP lists factors for clinics to consider during the assessment process.^31^ Questions related to these factors are also replicated in the HFEA’s proposed patient welfare history form that clinics may use when discharging their statutory responsibility.^32^ This requires patients to answer a short series of questions about convictions related to harming children and child protection measures, serious violence or discord within the family environment, mental or physical health conditions, the risk of any transmissible or inherited disorders, drug or alcohol problems, and any other aspects of the patient’s life or family history that may pose a risk of serious harm to any child the patient may have or anything which may impair the patient’s caring abilities.^33^

If the goal is to enable the effective allocation of scarce resources, s 13(5), framed and exercised in practice as a risk assessment, is an ineffective and inappropriate method to achieve it. For example, in 2019, it was reported that six Clinical Commissioning Groups (CCGs) in South East London were then operating on the basis of a policy that refused treatment to single women ‘because of the known disadvantage that providing assisted conception to a single woman would cause both the child and the mother’.^34^ Clinics were not, however, directed to reject patients during the pre-conception assessment in the event single status raised legitimate concerns about child welfare. Instead, a blanket policy refusing funding for assisted conception services to single women was adopted.

In support of this policy at the time, CCG documents cited an internal NHS document about fertility treatment for single women and same-sex couples.^35^ The report draws various conclusions supporting a case for excluding single women from treatment including the suggestion that ‘a sole woman is unable to bring about the best outcomes for children’,^36^ that they ‘exert less control on their children’,^37^ and are a ‘burden to society’.^38^ Given that the CCGs concerned did not cite any empirical evidence in support of their exclusion policies and referred only to this internal document, it is clear that value-laden judgments and subjective bias pertaining to welfare and the *ideal* family form are pervasive and arguably cross the boundary between moral assessment of a policy and moralizing.

Though a small number of patients may be refused treatment on welfare grounds (eg those with previous convictions for child abuse), it is more likely that the majority of patients will be accepted. As such, while the welfare principle may be responsive to assessing the potential risk of harm to any future children, it does nothing to assist with the allocation of resources among patients who satisfy the requirement, and it is fair to assume that, owing to individual economic circumstances and differing parenting practices, some patients will provide better outcomes for children than others.

Internationally, a number of jurisdictions have child welfare requirements that are either enshrined in legislation or contained within guidance issued by professional bodies. For example, like the UK, countries such as Canada,^39^ Australia,^40^ New Zealand,^41^ and Sweden (in cases of treatment with donor gametes)^42^ have also placed child welfare requirements in assisted reproduction on statutory footing. Though they differ slightly in form and emphasis with a number of different terms used to describe the protection offered (welfare as ‘paramount’^43^ or a bar to treatment^44^ versus ‘given priority’^45^ or ‘proper consideration’^46^), the substance of these measures is largely the same—consideration of a future child’s welfare.

A less formal approach can be seen in countries such as the USA, where consideration of child welfare is not legally mandated, but professional guidance to the sector advises clinics to take these considerations into account. For example, the Ethics Committee of the American Society for Reproductive Medicine (ECASRM) recently produced an updated guidance note in 2017 on child-rearing ability and the provision of fertility services.^47^ The advice is carefully worded such that consideration of ‘offspring welfare’^48^ is not required but recommended as a ‘valid consideration that fertility programs may take into account in accepting patients and providing services’.^49^ It is clear that as a complex fertility treatment for AUFI, considerations of child welfare are likely to be relevant to a large number of patients seeking UTx in many different countries. Exploring both the role and scope of such an assessment in the dual transplant-reproductive context is, thus, vital as research in this area continues apace.

### III.C. Preventing or Causing Harm? A Critique of the Pre-Conception Welfare Principle

Though different versions of a child welfare principle have been accepted in fertility law and policy internationally, criticism of welfare assessments in this context should not be discounted. This issue is particularly acute when considering the subjective nature of a welfare assessment in this context and the lack of a definition of welfare in the legislation mandating its consideration.

This lack of clear and definitive contextual framing in national iterations of the UK welfare principle means that screening will, in practice, typically be informed by individual practitioners’ own views on the family unit and the circumstances conducive to the promotion of child welfare. In turn, these factors themselves will typically be informed by the prevailing social and political norms of a given society. While the welfare principle may be framed as an objective tool to filter out ‘bad parents’, clearly, questions regarding what makes for a good life and whether particular patients are able to provide the circumstances conducive to one, are inherently normative. It is therefore reasonable to assume that the same case may be liable to generate different results in different clinics given the latitude and scope for disagreement between clinic staff on such matters.

Consider for example the case of single women seeking fertility treatment. In a study by Lee et al. in 2014, it was reported that ‘the treatment of single women was considered completely unproblematic at some clinics but, in others, single women still attracted particular scrutiny regarding their suitability as future parents’.^50^ This difference in approach is laid bare in interview extracts showing that for staff in some clinics, single status attracted consideration of factors such as the woman’s underlying motivations for seeking treatment (the example of a woman seeking to have a child in order to move into better social housing is provided), as well as the woman’s capacity to commit to personal relationships (a counselor questions whether the lack of a serious relationship for a number of years is reflective of commitment issues that may extend to a child).^51^ While it was reported that clinic staff ‘made a strong distinction between their own personal views and their professional obligations’,^52^ it is not clear, particularly in light of the latter example, that assessments were free from subjective bias and inconsistency on the part of the assessor.

Indeed, aside from the brief guidance contained in the HFEA CoP listing relevant factors for consideration,^53^ clinics are not directed in how to translate the information that is captured into a predictive assessment of the future family environment. This is problematic for two reasons. First among these is the value-driven nature of the assessment and the risk of subjective bias and assessor inconsistency. Second, as Waxman observes, clinics lack the relevant sociological or criminological expertise necessary to accurately predict whether a history of prior risk gives rise to reasonable concerns about potential future risk.^54^

Such concerns may prove even more pertinent in the UTx context where clinics in the UK are required not only to consider the welfare of any child who may be born as a result of treatment but also the welfare of any other child who may be affected by the birth. Here, care must be taken to ensure that patient scrutiny is not unduly influenced by authoritarian attitudes toward patient decision-making. For example, in a review of the selection criteria for the UK clinical trial Hammond-Browning suggests that ‘if account is taken of existing children of the family, the medical risks of this procedure further justify excluding women who are already mothers’.^55^ Though this claim is not explicitly based on the PCWP contained in the HFE Acts 1990 and 2008, the language of s 13(5)—taking into account existing children when determining who should have access treatment—is used.

Taking this seriously would not only preclude all women with existing children (through adoption, surrogacy or pregnancy prior to AUFI) from treatment,^56^ but may also lead to the conclusion that the birth of a second child should not be pursued due to the potential impact associated risk factors (such as organ rejection, cumulative immunosuppressant exposure, and adverse psychological risk factors associated with transplantation surgery)^57^ may have on the health of the patient and consequently the first-born child. There is, however, no empirical evidence to suggest that the welfare of children of transplant recipients is reduced by virtue of the patient’s transplant operation, recovery, and ongoing immunosuppressed state, and thus, it would be inconsistent to solely prevent UTx candidates from undergoing treatment on this basis. Furthermore, it would be unjustifiable to suggest that a patient’s status as parent and their responsibility for children provides reason to prohibit them from seeking and freely consenting to an operation. Consider the example of patients who seek elective, non-therapeutic cosmetic surgery requiring general anesthesia. Such surgery is, such as UTx, life-enhancing as opposed to life-saving and also poses physical and psychological risks. Yet, the existence of dependants alone is not generally considered to justify refusal to provide treatment.

Furthermore, in the UK, by virtue of the s 13(5) assessment, consideration of the welfare of an existing child is already required before fertility services can be provided. To suggest that the welfare of existing children should also play a role in patient selection as part of screening by the transplant team would be to extend the welfare principle far beyond its current remit and have some deeply counterintuitive implications. That said, the existence of other children *may* be a relevant consideration in policies governing access to UTx where difficult decisions must be made about the use of scarce public resources. In the NHS, for example, ‘childlessness’ is condition of access to publicly funded assisted conception services in the majority of Clinical Commissioning Groups in England.^58^ Those who have previously given birth to a child prior to the loss of their uterus or who have children through surrogacy or adoption may have a weaker claim to state resources than those who do not have any children, though a full discussion on appropriate funding criteria is beyond the scope of the present analysis.

Of the further criticisms that may be raised against the principle, for the purpose of this discussion it seems pertinent to outline the main three. The first objection to the PCWP is that the principle is discriminatory.^59^ This claim is based on the difference in treatment between infertile individuals seeking third party assistance in reproduction and their fertile counterparts. As Jackson,^60^ Blyth et al.,^61^ and Waxman^62^ (among many others) point out, fertile individuals are not required to undertake any such risk assessment prior to deciding to have children; neither are those traveling abroad for treatment in countries without a welfare principle nor those engaging in private at-home insemination arrangements. Infertile people exercising their reproductive autonomy, however, are uniquely subject to such scrutiny and for this reason the PCWP may be viewed as inherently discriminatory.

As Jackson contends, parental scrutiny transforms what would otherwise be a self-regarding decision—an exercise of the individual’s reproductive autonomy afforded morally and legally appropriate decisional privacy—into an other-regarding decision—a choice judged on the basis of the potential impact it may have on a hypothetical other.^63^ Though it has been suggested that the welfare principle contained in s 13(5) has been ‘liberalized’^64^ with the advent of reforms more inclusive of diverse family structures (the replacement of ‘need for a father’ with ‘supportive parenting’) and the introduction of a presumption to treat, as Waxman asserts, the current approach nevertheless ‘lacks parity with the unregulated world of natural conception’.^65^

The second criticism concerns the incoherent nature of the principle itself.^66^ In assisted reproduction, appeals are made to the welfare of any hypothetical child who does not yet exist. This is problematic where the proposed intervention will itself determine whether or not any particular child comes into existence.^67^ Welfare arguments determining the permissibility of an act, that is, the provision of treatment based on a hypothetical child’s welfare thus run afoul of Parfit’s ‘Non-Identity Problem’.^68^

In essence, a child born as a result of assisted reproduction cannot be said to be harmed by being created if it would not otherwise have been born, unless they have a life so poor as to be ‘not worth living’.^69^ If existence is preferable to non-existence (absent a life not worth living), or even if existence and non-existence are incommensurable, then it will surely almost never be against the interests of any child who might be born as a result of treatment, to be born. The exercise of judging familial and health circumstances to predict and project risk to welfare across an 18-year timeline of a non-existent child’s life is thus philosophically problematic. This clearly exposes the fundamentally different nature of pre-conception welfare assessments compared with those conducted in the case of adoption/legal disputes concerning existing children. In the latter case, the state owes a duty of care to the identifiable, existing child whereas the former concerns only the projected vision of *a* future child.

The third criticism leveled against the PCWP relates to the inconsistency of pre-conception considerations^70^ which, while forming the basis of decisions in the fertility context, are (i) suggested to be impossible in other areas of law, and (ii) do not apply to medical treatments falling outside of the HFE Act framework.^71^ As Jackson observes, the English courts’ resounding rejection of wrongful life negligence claims neatly illustrates this inconsistency.^72^

The claimant in a wrongful life claim is the person who would not otherwise have been born but for the defendant’s negligence, and the damage central to their claim is the life of the claimant itself. In essence, the claim is premised on the basis that the claimant’s non-existence is preferable to their existence in a disabled or impaired state arising from the defendant’s negligence. The Court of Appeal in the case of *McKay v Essex Area Health Authority* [1982]^73^ unanimously rejected such a claim, noting both the impossibility and artificiality of comparing existence with non-existence.^74^

In his judgment, Stephenson LJ went further, holding that a finding in favor of the claimant would be contrary to public policy given the sanctity of human life and would involve ‘valuing the life of a handicapped child as not only less valuable than the life of a normal child, but so much less valuable that it was not worth preserving’.^75^ As Jackson notes, obvious inconsistencies are present within this judgment, for it both confirms the courts’ hands to be tied due to the impossibility of comparing the states of being and not-being, and also appears to suggest that existence is to be preferred—a conclusion that Jackson notes is surely the product of some form of comparison.^76^

These differences in judicial reasoning notwithstanding, it remains the case that all three judgments stand in stark contrast to the mandated child welfare assessment in s 13(5). Though comparison between the states of existence and non-existence is deemed an impossible task with respect to the identification and quantification of damage for wrongful life, a comparison between these states is precisely what is required in the fertility context.^77^ This inconsistent legal approach to comparing existence with non-existence is problematic as it undermines the very foundation of the PCWP. Indeed, it is unclear how such comparison is thought to be unproblematic in one context and impossible in the other.

If it is the case that existence is preferable to non-existence unless a person’s life is of such poor quality as to not be worth living, then any consistency issues between the judgment in *McKay* and subsequent case law and the PCWP could be remedied to a degree by amending the wording of s 13(5). For example, though prospective parents are presumed to be supportive parents in the HFEA’s Code of Practice, this could be more strongly expressed in line with existence being preferable (absent a life not worth living) and placed on statutory footing.

A further, more practical, inconsistency can be found in the law’s approach to fertility-related treatments that do not fall within the remit of the HFE Act 1990 (as amended). Treatments falling outside this legal framework are not subject to the license conditions of the Act. This includes, for example, gynecological surgery to unblock the fallopian tubes, surgery to remove pelvic adhesions, surgery to remove endometriosis tissue, myomectomy (the removal of uterine fibroid(s)), and the provision of fertility drugs to boost ovulation.

Though patients may seek these treatments in order to facilitate conception and pregnancy—the same purpose for which patients may seek ARTs which do fall within the remit of the regulations—clinicians are not legally required to conduct any welfare-of-the-child assessment as a prerequisite to treatment provision. If scientific research into UTx advances to enable natural and spontaneous conception through sexual intercourse, whether this distinction between treatments can and should be maintained must be addressed. Though this issue is not a novel one given that the law already distinguishes between fertility-related treatments like those described above and treatment involving gametes and embryos, UTx brings it to the fore for two reasons. First, unlike other medical treatments such as gynecological surgery which may also have therapeutic indications, UTx has only one clear purpose—fertility—to treat AUFI, enabling the patient to give birth to her own child. And second, as a novel surgical treatment involving a number of potential risks to the health and well-being of the live donor, recipient, and developing fetus, such as IVF, UTx has, and continues to attract, wide-ranging scientific, ethical, and legal scrutiny that other fertility-related treatments have not.

Whether UTx should be categorized as treatment akin to other ARTs involving gametes and/or embryos with pre-conception welfare being a key consideration, or akin to fertility-related treatments such as other gynecological surgeries, is an issue that will need to be decided. In the UK, the requirement of IVF as part of the program of treatment presently masks the issue. However, this is not necessarily the case in countries without a legally mandated welfare assessment in the context of fertility treatment, and it certainly will not be the case here if scientific research into UTx in the future progresses to facilitate natural conception.

It may be objected that the criticisms explored above overstate concerns about the operation of the welfare principle in practice, and that, in reality, the assessments are no more than a minor inconvenience for patients required to fill out paperwork. However, I reject the idea that a discriminatory policy that serves to treat two groups of people (those able to conceive naturally and those requiring assistance) entirely differently is somehow less discriminatory because the effect of that discrimination is viewed as less disruptive by some.

Moreover, in recent times, evidence suggests that some CCGs (funding providers for ART services in England) have adopted over-zealous and disingenuous approaches to their statutory responsibility, utilizing it as a tool to ration treatment as opposed to a screening process genuinely concerned for child welfare.^78^ This in turn has led to inequalities of access to assisted reproduction across the UK.^79^ As such, the concerns articulated here about the continued operation of the s 13(5) assessment remain as, if not more, forceful today than when the law was first introduced.

Taking the above criticisms into account, it seems that a robust general case can be made against the imposition of the pre-conception welfare principle in the fertility context. However, political considerations regarding the protection of children must be borne in mind. In the UK, for example, notwithstanding legitimate concerns about discrimination and the incoherence of the task that the PCWP requires, given the emphasis on the paramountcy of child welfare in other areas of domestic and international law, it is likely to be the case that abolition of the principle is politically unrealistic. Having laid out important contextual information about the operation of the principle, I turn now to ask whether, given the novel and ethically contentious status of UTx, the application of the PCWP in this context raises any specific concerns.

## IV. SHOULD CHILD WELFARE AS A RELEVANT CONSIDERATION IN FERTILITY TREATMENT APPLY IN THE CASE OF UTERINE TRANSPLANTATION?

It should be noted at the outset that UTx for purposes other than having children has not been endorsed and is not currently being pursued given the associated health risks, the relative infancy of the procedure and the financial cost involved. An exploration of the risks and benefits of offering the procedure for other reasons is beyond the scope of this article but it seems fair to assume, on the basis of current data on the unknown/known health complications following UTx,^80^ that it is unlikely to be recommended. The assumed motivation for pursuing this treatment is clear throughout the literature.^81^ In the same way that IVF facilitates pregnancy in those unable to conceive, UTx facilitates gestation, enabling a woman to have her own child as a result of her own pregnancy; as such, it makes sense to think of UTx primarily as an addition to the list of ARTs.

As a procedure aimed at treating a particular form of infertility, it is understandable that appeals to precedent have been made seeking to highlight relevant similarities between UTx and other ARTs, given that their purpose is also the creation of children.^82^ As discussed in Section III, it is generally considered that welfare considerations are justified in the ART context given that the purpose of reproductive technologies is to create children. In this regard, an assessment of child welfare acts as a filter to screen out patients who, for health reasons or social factors relating to previous convictions or family violence, present an unacceptable level of risk to the welfare of a hypothetical child such that they should not be created and brought to birth. In other words, child welfare is employed as a threshold requirement determining access to fertility treatment. Before arguments regarding the use of welfare in the allocation of donor uteri can be discussed, it is necessary to examine whether, and in what circumstances, welfare as a relevant consideration in fertility treatment applies in the case of UTx. To answer this question, it must be determined whether the putative ethical similarities between UTx and existing fertility treatments are sufficiently grounded to justify assessment of child welfare in both cases. To this end, two scenarios must be explored: UTx as it is currently performed in light of existing scientific limitations and UTx as it may possibly be performed in the future.

### IV.A. Uterus Transplantation within the Current Limits of Medical Science

As explained previously, given that IVF is necessary for UTx to achieve its purpose—the treatment of AUFI resulting in the birth of a child—a s 13(5) assessment of the welfare of any child who may be born as a result of treatment and of any other child who may be affected by the birth, is mandated before IVF can be offered. In the case of standard assisted conception services without UTx, where consideration of the welfare principle is recommended by professional organizations such as the ASRM or legally required, it would be inconsistent, unjustified (and unlawful) to exempt UTx + IVF from the same consideration, all things being equal.

### IV.B. Welfare Considerations and Gynecological Surgery

The second scenario is currently a hypothetical one. It assumes that following UTx no reproductive medicine intervention is required and that recipients are able fall pregnant naturally through sexual intercourse, either because tubal functionality between the patient’s own oviducts and the uterine graft has been achieved, or because oviduct transplantation in addition to UTx was successful. Though treating AUFI would still be the goal of uterine transplant, as a transplant surgery that does not also require treatment involving gametes and/or embryos to achieve its purpose, the provisions of the HFE Act 1990 (as amended) would not apply.

Schedule 2, paragraph 1 to the 1990 Act lists a number of activities (a)—(g) that a license under the Act may authorize. These activities are not defined or listed according to their purpose, for example, fertility treatment or human reproductive research; instead, specific actions with regard to the use of human and/or animal embryos and gametes are outlined. As traditional ART treatments such as IVF, ICSI, intrauterine insemination, and PGT-M/SR (also known as pre-implantation genetic diagnosis (PGD)) involve the use of gametes and/or embryos, all fall within the listed activities. Gynecological surgery on the other hand falls outside the remit of the HFE Act’s licensing powers.

Given that surgery to facilitate conception and pregnancy does not involve the use of gametes or embryos, and is not itself listed as a licensable activity, the Act’s license conditions do not apply. This is so despite the fact that s 2(1) HFE Act 1990 (as amended) defines treatment services for the purposes of the Act as ‘medical, surgical or obstetric services provided to the public or a section of the public for the purpose of assisting women to carry children’. Applying the current law to these scenarios, it is clear that different versions of UTx will require different considerations of child welfare, that is, as a legal pre-condition to IVF treatment in the case of UTx + IVF versus no mandated consideration of welfare as a determinant of treatment at all where only UTx is required.

An easy solution to this conundrum would be to do nothing. However, given the media attention UTx attracts and the fact that it is solely performed for family-building purposes, this does not seem a fitting policy solution. Indeed, failure to even consider the issue may be regarded by some as a dereliction of duty on the part of policymakers. Alternatively, in seeking to promote consistency and treat like cases alike, it may be suggested that there is utility in adopting a purposive approach to fertility-related treatments—one that seeks to license gynecological surgery such as UTx depending on the aim or consequence of the treatment.

Note that I choose to focus this part of the analysis on gynecological surgery as opposed to other fertility-related interventions such as the provision of ovulation boosting drugs. This is because closer comparisons may be drawn between fertility-related surgeries and UTx in that the nexus of causation between the surgical intervention and the realization of pregnancy and childbirth is more obvious. Consider the following examples: A patient (X) may ovulate but not regularly and require drugs to stimulate regular follicular activity in the ovary. This drug intervention may cause an egg to be released that might not otherwise have been produced, and it may go on to be fertilized and develop into an embryo that implants into X’s uterus, continuing to develop leading to the birth of a child. However, as X’s condition was such that they did ovulate, albeit irregularly, there is also a chance that X’s ovary was always going to produce and release that same egg leading to the birth of a child. However, the chance of pregnancy and childbirth is close to, if not, zero where both of another patient’s (Y’s) oviducts are completely blocked meaning it would be impossible for sperm to reach and fertilize an egg. The same is true where Z was born without a womb meaning though they have ovaries and oviducts, sperm will never be able to reach and fertilize an egg, and there is no environment within which it can implant and develop. In both of these latter cases, a surgical intervention is required to give Y and Z greater chance of having a child as a result of their own pregnancies.

Licensing such treatments on the basis that parallels may be drawn between gynecological surgeries and regulated assisted conception services would align with the definition of treatment services provided in legislation where the purpose of ‘assisting women to carry children’^83^ is identified. As a licensed activity, gynecological surgery for this purpose would then be subject to the Act’s license conditions meaning the PCWP contained in s 13(5) would apply as a necessary pre-condition to treatment. There are, however, significant problems with such an approach.

First, for many gynecological surgeries, the restoration/improvement of fertility to assist women to carry children can be either (a) intentional or (b) a foreseen but unintended consequence. Group (a) cases, into which UTx falls, quite clearly align with the definition of treatment services in English law given the purpose is clearly identifiable and is the intended goal of treatment. As such, if it is determined that it is both desirable and justifiable to subject these treatments to greater regulatory oversight (which is not obvious), this may be achieved by amending the provisions of the HFE Act 1990 to specify treatments falling into Group (a) as activities requiring a license.

By contrast, group (b) cases arguably fall outside the scope of s 2(1) given that assisting women to carry children is not the *purpose* of treatment (but rather a foreseen but unintended consequence). If the pre-conception welfare principle was only applicable to group (a) cases this would create a flawed system of regulation since, for a number of treatments, doctors and patients could simply *claim* the particular surgery was a group (b) case and thereby avoid a welfare assessment. A possible solution to this scenario may be found in the presentation of the issue as a simple matter of statutory interpretation—reading down the legislation to consider that the purpose of group (b) cases satisfies the ‘purpose of assisting women to carry children’.^84^ Alternatively, one could seek amendment of the law to also add group (b) treatments as licensable activities. As a result, the PCWP would apply to both groups of cases. However, seeking to close the potential regulatory gap in this way would place an unduly onerous burden both on doctors providing, and patients seeking, treatment for the alleviation of particular symptoms caused by a particular medical condition.

To illustrate this point in more detail, consider the example of myomectomy (the removal of uterine fibroid(s)). This surgery may be performed solely to alleviate physical symptoms such as abdominal and lower back pain, constipation, heavy menstrual bleeding, pain during sex, and difficulty emptying the bladder.^85^ However, it may instead, or in addition to alleviating physical symptoms, be performed for the purpose of (a) intentionally restoring fertility where the presence of a fibroid may inhibit conception and pregnancy; or (b) fertility restoration may be a foreseen but unintended consequence of treating the patient’s particular symptoms. Patients in the latter case may either be undecided as to whether they might wish to have children at some point in the future or may genuinely have no intention of reproducing. As such, it would be both inappropriate and excessively onerous to require the patients of group (b) cases to satisfy non-medical criteria in the form of a child welfare assessment prior to offering surgery.

Furthermore, the issue is particularly acute when considering some of the factors that clinics consider relevant to the assessment process. For example, as noted in Section III single status is a factor that has attracted particular attention in deliberations about welfare previously. Compounding this is the emphasis NHS clinics more recently have placed on factors such as the stability of a relationship, the fact of cohabitation, the financial interdependence of the parties to a couple, smoking status, recreational drug use, and alcohol use, all of which are inextricably linked to the PCWP. Returning to the example of myomectomy, these wide-ranging social criteria are, for the most part, irrelevant in the context of decision-making about medical care for the treatment of uterine fibroids. Expanding the scope of the PCWP to apply in such cases would thus represent a significant and unjustified infringement of patient autonomy.

Second, in order to be consistent, and ensure that the requirements of some surgical treatments are not more burdensome than others, unless there exists just reason to distinguish between them, it would be necessary to identify and apply the PCWP to all gynecological surgeries that could be (a)-type, and possibly (b)-type cases. Indeed, while UTx and surgery to unblock the fallopian tubes for example are treatments that would clearly be classified as (a)-type cases, a number of other treatments may also be classified as (a)-type cases. It would thus both be inconsistent and unjust to apply such a framework to one or a number of these treatments and not all treatments all other things being equal.

Furthermore, assuming consistency in legal regulation is desirable, following this point to its logical conclusion suggests also identifying and licensing all possible medical and obstetric services for the purpose of assisting women to carry children given that there is no clear reason why surgical services should be singled out in isolation. In effect, this would mean that the PCWP would apply to all treatment services defined in s 2(1). This would clearly have a significant, disruptive, and intrusive impact on a wide range of medical treatments. There are also practical difficulties with such an approach concerning the issue of delineating the boundaries of what might count as ‘the purpose of assisting women to have children’. That is to say that any medical treatment provided to women of childbearing age that improves physical and/or mental health may also assist women to carry children. As a result, the potential list of applicable treatments may extend far beyond what might have been anticipated.

A related issue concerns current ambiguity present in guidance from the Regulator on the interpretation of s 13(5). While the legal position is clear, the HFEA Code of Practice, published to assist centers with their compliance with relevant legal provisions, is less so. Guidance Note 8 on the welfare of the child provision states that ‘[c]entres providing treatments that are not regulated by the HFEA but that fall within the definition of “treatment services”…may also find this guidance note *helpful*’.^86^ This suggests that clinicians may wish to address child welfare considerations prior to deciding to offer treatment to assist women to carry children that does not involve gametes or embryos. However, it stops short of recommending that clinicians *should* adopt this approach. Suggesting that a particular course of action may be ‘helpful’ is, ironically, largely unhelpful given the ambiguity in the chosen phrasing and the vast room this leaves open for the exercise of provider discretion. Accordingly, this does nothing to establish continuity between centers ensuring that like cases are treated alike, and this lack of consistency may potentially lead to wider inequalities of access to treatment.^87^

Ethically, it seems indefensible, *ceteris paribus*, to treat assisted reproductive technologies and other interventions such as UTx differently given that they both have relevantly similar ends and outcomes. However, this is precisely the distinction established by the legislative framework governing fertility services. Jackson observes that for Baroness Warnock, the chair of the enquiry into Human Fertilisation and Embryology that provided the foundation for current regulation, ‘it was axiomatic that “the good of the child… must be considered and taken into account”’.^88^ And yet, at no point during the Parliamentary debates leading to the enactment of the HFE Acts 1990 and 2008 was it proposed that the good of the child or parental fitness should be considered before clinicians provided other treatments to patients to improve their chances of conceiving.

Due to the current ambiguity in the HFEA’s guidance, and considering the particularly ethically controversial nature of UTx, should UTx evolve to no longer require ART, whether this distinction should be maintained must be properly debated and answered. I have sought to demonstrate that it would be unjust, unduly burdensome and practically difficult to license gynecological surgery like UTx and/or all treatment services for the purpose of assisting women to carry children. These arguments are also further compounded by the criticism of the PCWP explored at the beginning of this article in Section III.

## V. THE ROLE OF CHILD WELFARE IN THE ALLOCATION OF DECEASED DONOR UTERI

The equitable allocation of donor organs can be seen as having two distinct stages. First, the listing (threshold) stage ensures that only those meeting a particular set of agreed conditions are added to the transplant waiting list. And, second, the ranking (prioritization and optimization) stage enables clinicians to distinguish between listed candidates and identify who should receive a particular organ according to an agreed prioritization framework. Due to the unique nature of UTx and the condition it treats, unlike the allocation of more common transplants, the allocation of deceased and living non-directed uteri presents distinct and complex allocation challenges.^89^ This stems from the fact that AUFI is not a condition that comes in degrees of severity because ‘all those with it have an equal chance i.e. no chance of reproducing “naturally”’,^90^ as all patients lack the primary organ required for pregnancy and gestation.

Furthermore, the availability of transplantable uteri from deceased donors is likely to be scarce for a number of reasons. For example, of the prospective donors who die in circumstances where donation is possible, uteri from pre-menopausal, multiparous women are preferred, which significantly decreases the potential donor pool. In addition, consent to donation may be difficult to obtain given that as an organ excluded from new deemed consent legislation in England,^91^ Scotland,^92^ and Wales,^93^ express consent from the deceased’s next of kin will continue to be required throughout the UK (including Northern Ireland where the default donation system is opt-in) and family members may find it difficult to discern the patient’s wishes in relation to novel transplants and/or may find it difficult to ‘don the mental mantle’^94^ and separate their own views about what is arguably a sensitive organ from the views of the deceased.

Further, according to Kisu et al., brain death-induced systemic inflammation may negatively impact transplantation,^95^ and an increase in warm ischemia time owing to the fact that removal of the uterus should and will likely occur after the removal of vital organs may negatively affect both organ quality and functionality.^96^ As such, consideration of factors additional to those traditionally applied in other allocation frameworks (eg clinical health status, tissue type matching, time on waiting list, etc.) is required in order to rank and prioritize patients accordingly.

In addition to proposals^97^ relating to childlessness, age-related reproductive opportunity, motivation for seeking treatment, and the amount of ART treatment required, the assessment of child-rearing capacity (ie an assessment of child welfare) has been suggested as criterion to control access to donor uteri. For Bayefsky and Berkman, the establishment of a minimum standard for child-rearing capacity should form part of the minimum eligibility criteria for UTx (ie as a listing criterion).^98^ Bruno and Arora, on the other hand, have suggested that child-rearing capacity should be incorporated into the recipient ranking process (ie as a prioritization and optimization criterion) to address challenges in the fair allocation of deceased donor uteri.^99^ Though, in later commentary they acknowledge that separating listing and prioritization and utilizing welfare as a listing tool only may be preferable.^100^

What follows in the next two sections is an examination of the two proposals in favor of utilizing welfare in this way. In particular, I seek to provide a response to the question of what role the assessment of child welfare should play in the organ allocation process. Each stage of the process (listing and prioritization) will be examined separately in order to discern the potential merits and drawbacks of such an approach.

### V.A. Child Welfare as a Threshold Requirement Determining Eligibility for Transplant Listing

The first proposal in favor of assessing welfare is advanced by Bayefsky and Berkman, who recommend that minimum standards for child-rearing capacity^101^ should be developed which prospective patients should be required to meet before being listed as a potential uterus recipients.^102^ Defining the parameters of the assessment, the authors suggest that ‘criminality and financial stability’ constitute two key elements of this minimum standard.^103^ Though they are careful to note that the proposed financial standard ‘should not…preference wealthier women or families over less wealthy ones above a certain minimum’,^104^ they do not, however, seek to quantify or define this ‘minimum level of wealth’^105^ that a patient would be required to demonstrate in order to be listed for transplant. That said, the authors do envisage a mechanism by which patients may evidence they are able to meet this standard—through the provision of a ‘self-reported expense sheet along with documents to demonstrate the ability to cover basic monthly expenses’.^106^ Presumably, this would include the provision of documents including, but not limited to, bank statements, employment contracts, tenancy agreements, mortgage agreements, and utility bills.

To justify such an assessment of welfare, the authors appeal to precedent, seeking to draw relevant similarities between UTx and adoption, and UTx and assisted reproduction more generally.^107^ To this end, they point out that UTx shares similarities with adoption ‘in that the state must allocate a scarce resource and should do so responsibly’^108^ and with other fertility treatments in that UTx ‘is [also] a medical treatment’^109^ for infertility.

A number of issues may be raised in relation to the use of pre-conception welfare as a threshold criterion for transplant listing. First, the inclusion of social criteria such as child welfare may be contrary to ethical guidance on the development of allocation frameworks for selection and patient ranking. Horvat and Iltis note, for example, that the US Organ Procurement and Transplantation Network (OPTN) prohibits the inclusion of non-medical factors in allocation policies.^110^

In a guidance note updated in 2015, the OPTN states that ‘in particular, the social worth or value of individuals should not be considered, including social status, occupation, and so forth’.^111^ For Horvat and Iltis, it may well be the case that assessing child-rearing capacity is viewed as forming a judgment on the social worth of an individual or couple (the patient and their partner) with someone deemed unworthy of the social role of parent being refused access to the waiting list.^112^ Furthermore, guidance from the OPTN continues to note that, ‘allocation of organs based on social characteristics (such as race, socioeconomic class, gender) will conflict with the principle of justice’.^113^ It may thus be difficult to justify the requirement that patients should meet a minimum level of financial ability, particularly in countries with socialized welfare systems such as the UK.

Guidance in the UK on patient selection and allocation (ie placement on the waiting list and ranking priority) policies is less explicit about the inclusion of social judgments. However, it does state that relevant ‘policies need to balance a number of factors, some of which may be conflicting. Factors to be considered include clinical compatibility, equity, utility, benefit and fairness’.^114^ Notably absent from this description are non-medical social criteria.

Second, the appeal to precedent in this context to justify the imposition of the PCWP as a threshold criterion to restrict access to donor uteri is unpersuasive. Regarding the comparison with adoption, the justification seems to be that because the state has obligations to children for whom it is responsible, discharged by assessing the parental fitness of prospective adopters, the parental fitness of prospective UTx recipients should also be scrutinized given the causative responsibility the state has in creating children in this way.^115^ This is unconvincing for the same reason that appeals to adoption to justify the PCWP in ART are unconvincing—the subjects of those assessments are fundamentally different.^116^ Adoption concerns an *existing* child for whose welfare the state has become responsible. The PCWP in the UTx (and ART) context, however, concerns the welfare of a hypothetical child who may be born as a result of treatment, but whose very existence depends on the prospective patient passing the assessment and receiving the treatment in question.^117^

The two contexts are similar only in so far as there is some degree of state involvement in the facilitation of both practices. Whether that is sufficient to justify state scrutiny of prospective patients at the transplant listing stage of UTx is not, therefore, as self-evident as proponents of this position suggest. Regarding the analogy drawn between UTx and other ARTs, emphasis on the fact that UTx is a medical treatment seeks to position it as morally equivalent to other fertility treatments. This may turn out to be the case given that they share the same sole purpose—to enable the patient receiving treatment to fall pregnant and give birth to a child.

However, this raises more questions than the ‘problem’ it attempts to solve. For example, one might point out that there exists a number of medical and surgical treatments that may be performed for fertility purposes in addition to UTx that are not currently subject to the PCWP. As such, more work needs to be done to set out and justify *why* UTx should be singled out in this way. Bayefsky and Berkman justify their claims on the basis that

certain minimum criteria are necessary in cases of adoption and fertility treatment for three interrelated reasons:

(1) because it is better to promote the best interests of some future children, even if we cannot do so for all future children; (2) so that governments and physicians are not complicit in the mistreatment of future children; and (3) because of the social responsibility to act as sound stewards of a scarce resource, which requires that the state (or the OPTN, which has been empowered by the state) should promote (1) and avoid (2).^118^

The first reason offered is problematic for reasons already identified earlier in this paper regarding the philosophical problems with applying welfare considerations to pre-conception cases. For example, the non-identity problem implies that a person caused to exist by action X cannot be said to be harmed by that action unless their quality of life is so low as to not be worth living. As such, it is not clear that the best interests of some future children *are* promoted by preventing them from coming into existence (assuming existence is preferable to non-existence). Furthermore, this position is problematic because it completely disregards the interests that patients (and couples) requiring fertility assistance have in autonomous reproductive decision-making in order to further an end (promoting the best interests of some future children) that arguably cannot be achieved if we take seriously non-identity considerations.

Arguably there may be a difference in cases where embryos have already been created and the choice is between deciding upon the gestational environment for one particular embryo—the uterus of a surrogate or the recipient’s transplanted uterus following UTx. Whether identity questions arise in this context is complicated. It may be that this choice is person-affecting in such cases and depending on relevant evidence about risk and complications, it may be suggested that such an embryo is worse off being implanted in a donor uterus if this is deemed more risky than surrogate pregnancy.^119^ Nevertheless, my focus in this article is on cases where identity *will* be affected. This, I suggest, will be the majority of cases given that it is both highly unlikely that UTx patients will have already created embryos prior to being accepted for UTx treatment (eg for personal financial and/or public/insurer funding reasons), and it is highly unlikely that at present UTx centers would use such embryos in any event, meaning patients would be required to go through further IVF treatment to create new embryos for use in UTx pregnancy. This is because the majority of centers are performing the treatment in accordance with research protocols which specify a timeline for the sequential procedures comprising UTx (including IVF)^120^ and may also specify a minimum number of embryos required prior to transplantation and/or require additional embryo screening prior to freezing,^121^ which may not have been performed on any existing embryos patients have. As IVF is required to create (additional) embryos, and as many countries employ pre-conception welfare assessments as threshold tools to determine treatment provision, different embryos are thus likely to be created and transferred.

Regarding the second reason, to suggest an act that might cause a future person to exist renders the body/person who performed that act complicit in the mistreatment of someone who now exists is to stretch the boundaries of moral responsibility for all actions to the limit. Indeed, as Tonkens contends ‘with respect to actions performed in good faith…one is not morally blameworthy for the consequences of those actions that (a) one did not intend, (b) that one did not directly bring about oneself…and (c) where others are directly responsible for making sure that those consequences do not come about’.^122^ To illustrate this in another context, consider the example of a surgeon skilled in limb transplantation. We would surely not suggest that she would be complicit in the mistreatment of a patient’s wife simply because she performed the hand transplant surgery that provided a wife-beater with their instrument of mistreatment.^123^

Bayefsky and Berkman’s third justification for assessing welfare seems to be premised on the idea that scarce resources would have been wasted if fertility treatment leading to the birth of a child was provided to a patient who went on to abuse that child.^124^ If worries about the best use of scarce resources are to be taken seriously then this argument should extend to a number of fertility-related treatments (eg drugs to stimulate ovulation, surgery to unblock the fallopian tubes, etc.) without which the birth of a child may not, or would not, be possible. Why this should justify imposing the PCWP only in respect of UTx and other ARTs, is not, without more, clear. It may be suggested that assessing welfare in some cases, but not all, is more an issue of pragmatism than one of conceptual distinction such that it would simply not be feasible to license all prospective parents (including those who do not require any medical assistance). While this may be true, issues of practicality do not justify distinguishing between UTx/ARTs and other fertility-related interventions. For patients seeking both the former and latter services are equally positioned in that physicians have the same opportunity to conduct welfare assessments prior to conception while the patient is before them.

Further rejecting appeals to precedent, Tonkens points to what he identifies as an important difference between ARTs more generally and UTx making ‘ECASRM recommendations [on assessing offspring welfare] irrelevant in the latter context’.^125^ That difference, he explains, is the body likely to be responsible for welfare assessments, which in the context of UTx, he takes to be the body in control of the supply of donor organs—the state or a regulator contracted by the state such as the United Network for Organ Sharing (UNOS).^126^ This difference in the identity of the body with responsibility for assessment thus renders an appeal to the former context to justify the imposition of welfare in the latter, unpersuasive.^127^

Third, in countries with a legal or professionally endorsed welfare principle as a precursor to fertility treatment, the patient seeking UTx may potentially be subject to an assessment of welfare or child-rearing capacity *twice*. The relevant fertility clinic attended by the patient will assess relevant factors pertaining to child welfare, and, if the proposals noted above are to be accepted, this assessment would then be duplicated by the state organization responsible for listing transplant candidates. This is what other accounts, both supporting^128^ and rejecting^129^ the imposition of child welfare considerations in the uterus transplant listing process, unfortunately fail to acknowledge.

This atomistic view—conceiving of UTx as a transplant procedure, as opposed to a holistic view that recognizes the necessity of ART as part of the current^130^ program of UTx treatment, misses important potential problems and objections that may be raised by the prospect of double assessment. This is less problematic if both forms of assessment are the same—although assessors from two different specialties (fertility treatment providers and transplant physicians/the state as coordinator of national transplant listing) conducting the same or similar assessments may be viewed as inefficient and unduly onerous for the patient.

More problematic is the fact that there is no guarantee that the welfare assessments conducted for ART and listing purposes will be based on the same criteria or assessed according to the same standards if it is to be assessed twice. Indeed, in practice in both the UK and the USA, the PCWP is based on minimum standards. For example, as explained previously, HFEA guidance directs clinics to decide whether there is a risk of ‘*significant* harm’^131^ and to ‘presume that all prospective parents will be supportive parents’.^132^ Similarly, the ECASRM advises that fertility programs may deny treatment services where there are ‘*well-grounded* reasons that those patients will be unable to provide *minimally* adequate or safe care for offspring’,^133^ and that centers may provide services to those who would benefit from treatment ‘except when *significant* harm to a future child is *likely*’.^134^

Wall and Testa—two clinicians involved in the UTx trial at Baylor Medical Centre in Dallas, USA—observe that in the Baylor University Medical Centre (BUMC) trial, recipients underwent a five-stage evaluation process before being listed for UTx. This included: ‘(i) phone screening; (ii) objective medical and psychological evaluation; (iii) selection committee presentation; (iv) in vitro fertilization; and (v) final selection committee review’.^135^ They note that these listing requirements fulfill criteria relating to child-rearing capacity,^136^ for example. However, they unfortunately do not elaborate as to whether this was assessed as part of the IVF process in accordance with the ASRM’s Ethics Committee recommendations noted earlier in this piece, whether it was instead assessed by members of the multidisciplinary UTx team as part of the required psychological evaluation, or whether it was relevant to both stages of the listing process.

Furthermore, if offspring welfare was and is relevant to both stages of the process, it is not clear whether two assessments were conducted (and if so whether both applied the same method and standards) or whether the results of one informed or satisfied the other. While such considerations may not be an issue for patients being treated as part of a clinical trial where different specialties coalesce to form one multidisciplinary treating team following a specific trial protocol, these issues must be considered as centers begin to think about the translation of UTx to the clinical setting.

Bruno and Arora argue that the minimum threshold advanced by Berkman and Bayefsky including a criminal background check and financial stability ‘is too low’.^137^ Thus, they suggest that assessing welfare should involve a *comprehensive* assessment of child-rearing capacity similar to assessments undertaken of prospective adopters^138^—a seemingly more stringent and intrusive version of existing pre-conception welfare principles. Taking this suggestion for granted, the effect may be that patients are unduly burdened by the requirement to satisfy two different welfare assessments based on two different standards of child welfare. This may also result in unfair outcomes, particularly in private healthcare settings where health services are not integrated into a singular overarching system allowing more readily for a collaborative approach to healthcare treatment. For example, in countries with privatized healthcare, a patient could plausibly seek fertility treatment, pass relevant welfare checks, and go on to create embryos only to later find that they have been excluded from transplant listing for UTx after failing to meet the relevant welfare standard imposed by relevant transplant teams.

Fourth, the same criticisms raised in the ART context regarding the value-laden nature of the welfare assessment and the lack of sociological or criminological expertise apply also to the state/body in control of organ allocation. The very nature of assessments of child-rearing capacity, particularly comprehensive assessments involving ‘social work, psychology or similar evaluation’,^139^ likely require expertise beyond that possessed by those who would be responsible for evaluating child welfare in the context of transplant listing.

This issue may be mitigated by the contracting out of the assessment process to a private provider with the relevant knowledge and experience. However, this potential solution does nothing to justify the imposition of such assessments in this context in the first place. Related to this is the issue of ensuring clear and transparent factors relevant to any assessment are outlined, and that clear definitions of particular expected standards are provided for both patients and responsible assessors. For example, if it is accepted that patients should be required to meet a minimum level of financial stability, the threshold for this must be determined and accompanied by clear evidence-based explanations as to why a particular cut-off point was chosen.

Finally, it is suggested by Tonkens that screening potential UTx recipients for anticipated child-rearing ability, and not also screening other relevantly similar family builders, amounts to unjustified discrimination.^140^ This is a general argument that applies to the context of assisted reproduction more generally discussed in Section III.^141^ Essentially, it does not follow from the fact an individual or couple requires assistance to conceive (and gestate) that concerns for child welfare are more acute, thus necessitating scrutiny pre-conception.^142^

### V.B. Child Welfare and Organ Optimization: Why Parenting Capacity Should Not Determine Priority

While the number of women who seek UTx may be relatively small overall, it is nevertheless likely that demand for a uterus will outstrip the supply of uteri available for donation. This is particularly problematic given that the considerations traditionally used to inform prioritization algorithms are unhelpful in the case of UTx. As noted above, it is not possible to prioritize patients on the basis of considerations such as medical need or the severity of the patient’s condition given that all patients have the same need for a uterus in order to gestate a pregnancy. As such, in addition to tissue type matching, it will be necessary for other relevant factors such as patient age and position within a determined range of childbearing years^143^ to feed into allocation algorithms in order to determine patient priority for an organ. Building on the work of Bayefsky and Berkman who propose using a minimum standard for child-rearing capacity as an eligibility criterion for UTx listing, Bruno and Arora^144^ have previously endorsed child-rearing capacity as a relevant prioritization factor and suggest further expansion of the scope of any assessment.^145^

Noting that the previous proposal was based on a *minimum* standard for child-rearing capacity, Bruno and Arora instead recommend a ‘*comprehensive* assessment of the ability to rear a child’.^146^ To this end they propose that a social work, psychology or similar evaluation should be conducted prior to the patient being listed for transplant.^147^ Like the previous authors, they are careful to note that such an assessment should not be based on one’s own social value judgments about good parenthood or motherhood.^148^ Instead, they adopt language borrowed from child law (relating to children after birth) and argue that ‘rather, the best interest and safety of the future child be paramount’.^149^

At this point a minor reflection on the use of language must be observed: the suggestion by Bruno and Arora that child welfare should be paramount effectively elevates child welfare as the primary concern in decisions about both patient selection/exclusion in UTx.^150^ This is problematic given that as it currently applies in the regulation and practice of ARTs more generally, the legally mandated (UK) or professionally recommended (US) PCWP is *not* paramount. Indeed, in both of these cases, the PCWP involves a determination of whether a risk of significant harm or neglect to a future child exists. In the UK, child welfare shall, however, ‘be the court’s paramount consideration’ when determining any question with respect to (a) the upbringing of a child; or (b) the administration of a child’s property or any income arising from it.^151^

Clearly, the two contexts are very different—one concerns welfare in the context of family proceedings in respect of existing children and the other concerns the welfare of a hypothetical child in fertility treatment—hence the different welfare standard applied. It would therefore be unusual, inconsistent and unfair for child welfare to occupy this position solely in relation to UTx establishing a higher bar for patients requiring IVF in UTx to meet than those requiring fertility services.^152^

In their work proposing factors to consider in the allocation of donor uteri, Bayefsky and Berkman reject the use of child welfare in patient ranking on the grounds that such an undertaking would be ‘burdensome and morally questionable’.^153^ This position seems correct and indeed, it is one to which Bruno and Arora acquiesce in later commentary where they comment that separating listing and prioritization would address concerns about including parental fitness into existing allocation frameworks and regulation.^154^ However, I would go further than this and caution against using child welfare considerations in this way on the grounds that it may lead to (i) damaging and unjustly discriminatory conclusions that constitute an unjust infringement of the reproductive autonomy and freedoms of prospective patients and (ii) the creation of inequalities between those seeking treatment.

For child welfare to be useful as a tool to optimize the allocation of donor organs among the list of eligible patients, the assessment would need to be more than a threshold requirement (as it operates in fertility law and policy). Instead, it would need to function to enable the assessor to score prospective patients (and their partners) on child welfare grounds, that is, the patient with the highest score would be the one deemed best able to meet the welfare needs of a future child. In a patient ranking algorithm, this would presumably mean that the patient with the highest child welfare score would receive more points, and thus higher priority on the transplant waiting list. However, the problem inherent in this hypothetical process is the fertile ground it paves for unjustified discrimination against particular patients or particular groups of patients.

The same criticisms explored elsewhere in this paper regarding the risk of subjective bias and assessor inconsistency apply equally, if not more forcefully, in this context. This concerns the difficulty with objectively defining parenting qualities most conducive to child welfare and the characteristics reflective of a supportive, welfare-promoting environment. Further difficulty is raised by the prospect of applying to that definition an appropriate scale that equates particular criteria being met by the prospective parent(s) with a particular number of points. This has the potential to be significantly more burdensome and unjustly discriminatory than the use of child welfare as a threshold tool as it both implies a deeper level of scrutiny, and leaves open considerably more room for reasonable disagreement as to which qualities are more important to promote child welfare and which prospective parents best display these qualities.

In effect, this would require those responsible for managing the transplant listing and prioritization process to compare and rank patients according to imagined futures and perceived ability to meet different thresholds of a particular parenting standard. This could result in the marginalization of particular patients or patient groups due to biased listing procedures, for example, single women who, though they may be judged to present no significant risk of harm to a future child and able to provide minimally adequate or safe care, may not fit a projected ideal of the *best* parent(s) able to provide a future child with the *best* life, leading to their de-prioritization on the transplant waiting list as a result.^155^

Added to this, and as Horvat and Iltis observe in the US context, the inclusion of social factors like welfare-screening outcomes to inform prioritization frameworks would arguably be contrary to ethical guidance on patient selection and allocation.^156^ This would also be the case in the UK where guidance from NHS Blood and Transplant (the body responsible managing donation and transplantation infrastructure in the UK) provides that ‘allocation must not be on, for example, the basis of ethnicity, age, gender, disability, *life-style*, and *perceived value to society* or ability to pay’.^157^

## VI. CONCLUSION

The PCWP arguably remains as controversial today as it was when first introduced as a legal requirement in the UK. While the passage of time has not served to diminish any of the valid criticisms that may be leveled against it, it has served to entrench its operation in law and policy. In practical terms, and from a political perspective, this means that it is likely the principle will endure. As such, novel fertility treatments, and their potential future development, must be examined in light of existing law and policy. This is particularly important where potential scientific advances may lead to new fertility treatments falling outside the scope of regulatory systems governing current reproductive technologies. Proper debate must therefore be had about how the law should respond to new treatments and what, if any, checks and balances they ought to be subject to.

This article has examined the role of the PCWP in our ethical and legal reasoning about UTx. The analysis revealed that a number of ethico-legal issues with potentially unjust discriminatory and unfair consequences may arise, depending on the policy significance attributed to assessing welfare in the provision of UTx treatment. These issues may neatly be summarized as (i) a lack of consistency caused by potential gaps in regulation and (ii) the unjustified expansion of welfare assessments.

At present, the fact that IVF is required as part of the program of UTx treatment serves to obscure, or at least acts as an unsolicited and fragile solution to, a number of issues that would otherwise arise. As natural conception is not currently possible in UTx, all patients seeking it in the UK will be required to undergo a welfare of the child assessment prior to creating embryos via IVF. However, this status quo will only be maintained for as long as IVF continues to be required. If further research into UTx is pursued and it becomes surgically feasible to connect the uterine graft to the patient’s or transplanted oviducts, pregnancy arising from spontaneous natural conception may then be possible. As a consequence, and without the requirement for IVF, UTx would fall into a regulatory gap currently occupied also by fertility-related treatments that do not involve gametes or embryos, and thus do not require a license.

While this potential issue is not novel for that reason, I have suggested that the possibility that UTx may develop in this way forces us to re-consider whether the current distinction between treatments based on the involvement, or not, of gametes and embryos can, and should be, maintained. This consistency issue is one that regulators must be prepared to grapple with in advance of potential UTx developments that will direct fresh attention to questions policymakers have thus far been content to ignore. I have signaled the regulatory difficulty dealing with this question may raise, setting out why it would not be desirable or feasible on grounds of political expediency to amend the current legislation governing human reproduction to include all fertility-related treatments. Further, though this would provide both legal consistency and certainty, it would do so at the expense of unjustly infringing patient reproductive autonomy. For example, patients who seek treatment for which fertility restoration may be a foreseen but unintended consequence, and who may be undecided about having children, may end up being required to pass welfare screening in order to be relieved of the burden of their condition. Such intrusive scrutiny as a condition of access to medical treatment is clearly undesirable and would further be practically difficult (if not impossible) to implement. That said, a case for the alternative solution—the singling out of UTx for specific regulation mandating welfare screening as a condition of access—is no more obvious. Anyone who believes the PCWP should be applied solely to UTx in this context must therefore not only defend s 13(5) against extensive existing critique, but also the decision to differentiate UTx from other fertility-related treatments, and justify the requirement for an additional layer of state scrutiny in the former but not the latter case.

Whether pre-conception welfare considerations should have a role in the allocation of deceased donor uteri is an important question that must be addressed by policymakers and clinicians devising treatment protocols both at the research stage and when considering the clinical translation of UTx. Based on the examination presented in this article, proposals to utilize child welfare as a threshold tool to determine patient access and listing should be rejected. This is because the inclusion of social factors may be contrary to ethical guidance on patient selection and allocation, and it would arguably be unfairly burdensome to potentially subject UTx recipients to welfare assessments twice (as a filter to access IVF *and* as part of a transplant listing decision). Furthermore, the application of child welfare measures to inform patient allocation algorithms should also be explicitly rejected by clinicians involved in UTx treatment and state bodies controlling the supply of donor uteri. The inclusion of social factors would not only arguably be against ethical guidance but may have unjustly discriminatory consequences and unjustly infringe the reproductive autonomy of prospective patients.

## Footnotes

1 Benjamin P. Jones et al., *Uterine Transplantation: Past, Present and Future*, 123 BJOG 1434 (2016).

2 Laura O’Donovan, *Pushing the Boundaries: Uterine Transplantation and the Limits of Reproductive Autonomy*, 32 BIOETHICS 489 (2018).

3 Liza Johannesson and others, *Life Underneath the VCA Umbrella: Perspectives from the US Uterus Transplant Consortium,* 21 AM. J. TRANSPLANT. 1699, 1700 (2021).

4 At the time of writing in July 2022, there are 21 UTx studies registered on ClinicalTrials.Gov at institutions in Europe, North America, South America, and Africa, one study is registered on the Australian and New Zealand Clinical Trials Registry and trials are also reported to be taking place across Asia. See ClinicalTrials.Gov, Search Results, https://clinicaltrials.gov/ct2/results?pg=1&load=cart&id=NCT02409147+OR+NCT03581019+OR+NCT03138226+OR+NCT01844362+OR+NCT03284073+OR+NCT03277430+OR+NCT04026893+OR+NCT04615221+OR+NCT02656550+OR+NCT03307356+OR+NCT02987023+OR+NCT04249791+OR+NCT03590405+OR+NCT02573415+OR+NCT04314869+OR+NCT03252795+OR+NCT04244409+OR+NCT02741102+OR+NCT03048396+OR+NCT02388802+OR+NCT03689842 (accessed July 10, 2022); ANZCTR: Trial Registration: ACTRN12619000808145,https://www.anzctr.org.au/Trial/Registration/TrialReview.aspx?id=377613&isReview=true (accessed July 10, 2022); Iori Kisu and others, *Current Progress in Uterus Transplant Research in Asia,* 8 J. CLIN. MED. 245 (2019).

5 See Sheryl L. Lacey et al., *Child Interests in Assisted Reproductive Technology: How is the Welfare Principle Applied in Practice?* 30 HUM. REPROD. 616 (2015) discussing how pre-conception child welfare is considered in countries including Australia, Canada, United Kingdom, and New Zealand. See also Ethics Committee of the American Society for Reproductive Medicine, *Child-rearing Ability and the Provision of Fertility Services: An Ethics Committee Opinion,* 108 FERTIL. STERIL. 944 (2017).

6 Bethany Bruno and Kavita Shah Arora, *Uterus transplantation: The Ethics of Using Deceased versus Living Donors*, 18 AJOB 6 (2018).

7 Cesarean section is the recommended modality of delivery in UTx due to the particular anatomical considerations of a transplanted uterus. Note the term abdominal birth is deliberately chosen for two reasons: (i) to demonstrate that birth via cesarean section is an equally valid (though not necessarily preferred) birth experience and (ii) to more accurately reflect the lived experience of this method of delivery.

8 Note the term fallopian tube and oviduct refer to the same anatomical structure in the biologically female anatomy and will henceforth be used interchangeably.

9 Jones et al., *supra* note 1, at 1434.

10
Deb Kumar Boruah et al., *Spectrum of MRI Appearance of Mayer-Rokitansky-Kuster-Hauser (MRKH) Syndrome in Primary Amenorrhea Patients*, 11 J. CLIN. DIAGN. RES. TC30 (2017).

11 Comprising also the cervix, a cuff of vagina, the surrounding ligamentous and connective tissues as well as the major blood vessels to the level of the internal iliac vessel. See Benjamin P. Jones et al., *Uterine Transplantation in Transgender Women*, 126 BJOG 152 (2019).

12 Mats Brännström et al., *Experimental Uterus Transplantation*, 16 HUM. REPROD. UPDATE 329, 332 (2010).

13 Brännström, *supra* note 12, at 332.

14
*Id*.

15 See Georg Sillo-Seidl, *The First Transplantation of a Fallopian Tube of Frozen Material in Woman*, 20 INT. J. FERTIL. 10 (1975); Carl Wood et al., *Microvascular Transplantation of the Human Fallopian Tube*, 29 FERTIL. STERIL. 607 (1978). See also Wafa Fageeh et al., *Transplantation of the Human Uterus: Case Report*, 76 Int. J. GYNECOL. OBSTET. 245 (2002) for an account of the first modern human uterine transplant in Saudi Arabia in April 2000. This involved the transplantation of the uterus and oviducts. Hysterectomy due to acute vascular thrombosis was required 99 days after transplant, but both fallopian tubes were said to have remained pink and viable.

16 Human Tissue (Permitted Materials: Exceptions) (England) Regulations 2020, SI 2020/521, reg 2(2)(dd).

17 Section 13(5) Human Fertilisation and Embryology Act (HFE Act) 1990 (as amended).

18 That is, as a treatment for natal females diagnosed with AUFI.

19 AMEL ALGHRANI, REGULATING ASSISTED REPRODUCTIVE TECHNOLOGIES: NEW HORIZONS (2018). However, I suggest that this is less likely in the context of NHS fertility treatment as a number of Clinical Commissioning Groups (bodies responsible for the funding of particular treatments such as IVF in defined geographical locations) have recently updated their assisted conception commissioning policies to confirm that they will not commission assisted conception services where the intention is that the embryo will be carried in a transplanted uterus on the grounds of cost effectiveness (see Blackpool Clinical Commissioning Group and Flyde and Wyre Clinical Commissioning Group, *Policies for the Commissioning of Healthcare: Policy for Assisted Conception Services* (2020) https://www.fyldecoastccgs.nhs.uk/document/policy_for_assisted_conception_services_v2-0-docx/ (accessed July 10, 2022).

20 Department of Health, *Review of the Human Fertilisation and Embryology Act: Proposals for revised legislation (including establishment of the Regulatory Authority for Tissue and Embryos)* (2006) https://assets.publishing.service.gov.uk/government/uploads/system/uploads/attachment_data/file/272391/6989.pdf (accessed July 10, 2022).

21 Sections 5–10 of the HFE Act 1990.

22 Schedule 2, para 1(1)(a)-(g) of the HFE 1990 (as amended).

23 Section 14(2)(b) of the HFE Act 2008 amending the HFE Act 1990.

24 See Guardianship of Infants Act 1925; Guardianship of Minors Act 1971; Children Act 1989.

25 See, for example, HL Deb 13 March 1990, vol 516, Col 1487.

26 Emily Jackson, *Conception and the Irrelevance of the Welfare Principle*, 65 MOD. L. REV. 176, 176 (2002).

27 See HL Deb 6 March 1990 vol 516, Cols 1053–115 and HC Deb 2 April 1990 vol 170, Cols 914–85.

28 Human Fertilisation and Embryology Authority, *Improved Welfare Checks System will be Better, Fairer and Clearer for Fertility Patients, GPs and Clinics*, Press release, Nov. 2, 2005 (London, HFEA 2005).

29 Human Fertilisation and Embryology Authority, *Code of Practice 8th edn* (2009), 96, https://www.hfea.gov.uk/media/2062/2017-10-02-code-of-practice-8th-edition-full-version-11th-revision-final-clean.pdf (accessed July 10, 2022).

30 Human Fertilisation and Embryology Authority, *Code of Practice 9th edn* (2021), https://portal.hfea.gov.uk/media/ihkjnfqq/2022-07-01-code-of-practice-2021.pdf, at para 8.15, accessed (July 5, 2022) (emphasis added).

31
*Id*. at para 8.14.

32 Human Fertilisation and Embryology Authority, *Patient History Form*, https://www.hfea.gov.uk/media/2791/welfare-of-the-child-form-v3.pdf (accessed July 5, 2022).

33
*Id*.

34 Gabriel Pogrund, *NHS Trust Denies Single Women IVF Treatment*, THE TIMES (London, Aug. 18, 2019), https://www.thetimes.co.uk/article/nhs-trusts-deny-single-women-ivf-treatment-gs9b7qxbt, (accessed July 5, 2022).

35
*Id*. Note that this newspaper article reproduced excerpts of the report in question which was removed from its host website shortly after the publication of the news article.

36
*Id*.

37
*Id*.

38
*Id*.

39 Assisted Human Reproduction Act 2004.

40 South Australia Assisted Reproductive Treatment Act 1988; Victoria Infertility Treatment Act 1995; Western Australia Human Reproductive Technology Amendment Act 2004; New South Wales Assisted Reproductive Technology Act 2007.

41 Human Assisted Reproductive Technology Act 2004.

42 SFS (The Swedish Statute Book), 2006. 351 Lagen om genetisk integritet [Act on genetic integrity].

43 S 4A of the Assisted Reproductive Treatment Act 1988 (South Australia).

44 S 13(5) of the Human Fertilisation and Embryology Act 1990 (as amended) (UK).

45 C 2, s 2(a) of the Assisted Human Reproduction Act (Canada).

46 S 4(1)(d)(iv) of the Human Reproductive Technology Act 1991 (Western Australia).

47 Ethics Committee of the American Society for Reproductive Medicine, *Child-Rearing Ability and the Provision of Fertility Services: An Ethics Committee Opinion* 108 FERTIL. STERIL. 944 (2017).

48
*Id*.

49
*Id*. at 947.

50
Ellie Lee et al., *Assessing Child Welfare under the Human Fertilisation and Embryology Act 2008: A Case Study in Medicalisation?*, 36 SOCIOL. HEALTH & ILLN. 500, 509 (2014).

51
*Id*. at 511.

52
*Id*. at 509.

53 Human Fertilisation and Embryology Authority, *supra* note 30.

54 Sacha Waxman, *Applying the Pre-conception Welfare Principle and the Harm Threshold: Doing More Harm Than Good?*, 17 MED. LAW INT. 134, 143 (2017), at 139.

55 Natasha Hammond-Browning, *UK Criteria for Uterus Transplantation: A Review*, 126 BJOG 1320, 1323 (2019).

56 A stronger argument for this may be founded on resource allocation grounds and an appeal to precedent.

57 See O’Donovan, *supra* note 2, for an account of the possible physical and psychological risks uterine transplantation presents for the live donor, recipient, and resulting child.

58 British Pregnancy Advisory Service, *BPAS Investigation into the IVF Postcode Lottery: An Examination of CCG Policy for the Provision of Fertility Services*, https://www.bpas.org/media/3369/bpas-fertility-ivf-postcode-lottery-report.pdf (accessed July 5, 2022).

59 See Gillian Douglas, *Assisted Reproduction and the Welfare of the Child*, 46 CURR. LEG. PROB. 53, 69 (1993).

60 Jackson, *supra* note 26.

61 Eric Blyth et al., *Welfare of the Child Assessments in Assisted Conception: A Social Constructionist Perspective*, 26 J. REPROD. INFANT PSYCHOL. 31, 37 (2008).

62 Waxman, *supra* note 54.

63 Jackson, *supra* note 26, at 182..

64 Ellie Lee et al., *Assessing Child Welfare Under the Human Fertilisation and Embryology Act: The New Law* (2012) https://blogs.kent.ac.uk/parentingculturestudies/files/2012/06/Summary_Assessing-Child-Welfare-final.pdf (accessed Dec. 6, 2021).

65 Waxman, *supra* note 54, at 156.

66 See Jackson, *supra* note 26, at 196–199 and Waxman, *supra* note 56, at 146–148.

67 I. Glenn Cohen, *Regulating Reproduction: The Problem with Best Interests* 96 MINN. L. REV. 423 (2011).

68 DEREK PARFIT, REASONS AND PERSONS (1984).

69
*Id*.

70 EMILY JACKSON, REGULATING REPRODUCTION: LAW TECHNOLOGY AND AUTONOMY (2001); Eric Blyth and Chris Cameron, *The Welfare of the Child: An Emerging Issue in the Regulation of Assisted Conception*, 13 HUM. REPROD. 2339 (1998).

71 Jackson, *supra* note 26, at 192, 196–199.

72
*Id*.

73
*McKay v Essex Area Health Authority* [1982] QB 1166.

74
*Id*. at [1184] (Stephenson LJ), [1192] and [1193] (Ackner LJ) of the judgment.

75
*Id*. at [1180] (Stephenson LJ).

76 Jackson, *supra* note 26, at 196–197.

77
*Id*.

78 See, for example, *NHS Bradford District and Craven CCG, Access to Fertility Treatment: Commissioning Policy Document: Yorkshire and Humber*, 14, http://www.bradfordcravenccg.nhs.uk/wp-content/uploads/2020/03/2020-04-01_Policy_Fertility_BDC_CCG.pdf (accessed June 6, 2022); Devon Clinical Commissioning Group, *Assisted Conception*, 6, https://devonccg.nhs.uk/?s=IVF (accessed June 6, 2022); and Kernow Clinical Commissioning Group, *Commissioning Policies 2019/20*, 58, http://doclibrary-kccg.cornwall.nhs.uk/DocumentsLibrary/KernowCCG/IndividualFundingRequests/Policies/CommissioningPolicies.pdf (accessed June 6, 2022).

79 For more details about the extent of current policy inequalities, see British Pregnancy Advisory Service, *supra* note 58.

80 See O’Donovan, *supra* note 2; Benjamin P. Jones and others on behalf of the Royal College of Obstetricians and Gynaecologists, *Uterine Transplantation. Scientific Impact Paper No. 65*, 128 BJOG e51 (2021).

81 See Anjana Nair et al., *Uterus Transplant: Evidence and Ethics*, 1127 ANN. NY ACAD. SCI. 83 (2008); Giuliano Testa and Liza Johannesson, *The Ethical Challenges of Uterus Transplantation*, 22 CURR. OPIN. ORGAN TRANSPLANT. 593 (2017); Mats Brännström et al., *Uterus Transplantation: A Rapidly Expanding Field*, 102 TRANSPLANTATION 569 (2018); and Richards et al., *supra* note 9.

82 See Michelle J. Bayefsky and Benjamin E. Berkman, *The Ethics of Allocating Uterine Transplants*, 25 CAMB. Q. HEALTHC. ETHICS 350, 359 (2016).

83 Section 2(1) of the HFE Act 1990 (as amended).

84
*Id*.

85 National Health Service, *Overview: Fibroids*, https://www.nhs.uk/conditions/fibroids/ (accessed July 10, 2022).

86 Human Fertilisation and Embryology Authority, *supra* note 30, at para 8.1 (emphasis added).

87 Note that I am in the process of gathering empirical research data derived from Freedom of Information requests sent to all public fertility providers in England to determine whether any clinics have acted upon this advice from the Regulator and conducted welfare of the child assessments before providing fertility-related treatments that do not involve gametes and/or embryos.

88 Jackson, *supra* note 26, at 180.

89 Laura O’Donovan et al., *Ethical and Policy Issues Raised by Uterus Transplants*, 131 BMB 19, 24 (2019).

90
*Id*.

91 Human Tissue (Permitted Materials: Exceptions) (England) Regulations 2020, SI 2020/521.

92 Human Tissue (Excepted Body Parts) (Scotland) Regulations 2020, SSI 2020/388.

93 Human Transplantation (Excluded Relevant Material) (Wales) Regulations 2015, SI 2015/1775 (W 247).

94 TOM F. BEAUCHAMP AND JAMES F. CHILDRESS, PRINCIPLES OF BIOMEDICAL ETHICS (1994), 171.

95 Iori Kisu et al., *Current Status of Uterine Transplantation in Primates and Issues for Clinical Application*, 100 FERTIL. STERIL. 280 (2013).

96
*Id*. at 286–288.

97 Bruno and Arora, *supra* note 6.

98 Bayefsky and Berkman, *supra* note 82.

99 Bruno and Arora, *supra* note 6.

100 Bethany Bruno and Kavita Shah Arora, *Uterus Transplantation: Response to Open Peer Commentaries on the Ethics of Using Deceased Versus Living Donors*, 18 AJOB W6 (2018**)**; Anji Wall and Giuliano Testa, *Living Donation, Listing and Prioritization in Uterus Transplantation*, 18 AJOB 7 (2018).

101 It should be understood that assessment of child-rearing capacity serves to protect the welfare of any child who may be born as a result of treatment and throughout this analysis will also be referred to as a child welfare assessment. This is due to jurisdictional differences in terminology referring to the same concepts.

102 Bayefsky and Berkman, *supra* note 82, at 359.

103
*Id*.

104
*Id*.

105
*Id*.

106
*Id.* at 360.

107
*Id.* at 359–360.

108
*Id*. at 360.

109
*Id*.

110 Margaret Horvat and Ana Iltis, *What Are Good Guidelines for Evaluating Uterus Transplantation?*, 21 AMA J. ETHICS E988, 992 (2019).

111 Organ Procurement and Transplantation Network, *Ethical Principles in the Allocation of Human Organs*, https://optn.transplant.hrsa.gov/resources/ethics/ethical-principles-in-the-allocation-of-human-organs/ (accessed June 10, 2022).

112 Horvat and Iltis, *supra* note 110, at 992.

113 Organ Procurement and Transplantation Network, *supra* note 111.

114 NHS Blood and Transplant, *Introduction to Patient Selection and Organ Allocation Policies*, https://nhsbtdbe.blob.core.windows.net/umbraco-assets-corp/4955/introduction_to_selection_and_allocation_policies.pdf (accessed June 10, 2022).

115 Responsibility in this sense may be said to arise from a states’ direct funding of UTx to enable recipients to have children in this way, or it may arise from the fact of permissive regulatory regime/reproductive policy that facilitates the provision and social acceptance of UTx.

116 See Jackson, *supra* note 26, at 195 and Ryan Tonkens, *Misusing Uteruses? Childrearing Capacity and Access to Transplantable Wombs*, 29 BIOETHICS 389 (2020).

117 See Cohen, *supra* note 67.

118 Bayefsky and Berkman, *supra* note 82, at 360.

119 Judith Daar and Sigal Klipstein, *Refocusing the Ethical Choices in Womb Transplantation*, 3 J. LAW BIOSCI. 383 (2016); John A. Robertson, *Impact of Uterus Transplant on Fetuses and Resulting Children: A Response to Daar and Klipstein*, 3 J. LAW BIOSCI. 710 (2016), at 713–714.

120 See ClinicalTrials.Gov, *supra* note 4.

121 Rhea Chattopadhyay et al., *Preimplantation Genetic Testing for Aneuploidy in Uterus Transplant Patients,* 15 THER. ADV. REPROD. HEALTH 1 (2021).

122 Tonkens, *supra* note 116, at 111.

123 I thank my colleague Dr Nicola Williams for this useful example.

124 See Tonkens, *supra* note 116, also on this point who conceptualizes this issue as the waste, or misuse of scarce medical resources, ie the transplant.

125
*Id*, 109.

126
*Id*.

127
*Id*.

128 Bayefsky and Berkman, *supra* note 82; Bruno and Arora, *supra* note 6; Anji Wall and Giuliano Testa, *Living Donation, Listing and Prioritization in Uterus Transplantation*, 18 AJOB 7 (2018).

129 Tonkens, *supra* note 116.

130 Note that the issue of double assessment would no longer be relevant if UTx research progresses to facilitate natural conception in the future. Instead, the issue would become one of whether UTx should be subject to welfare assessments in its own right as a transplant surgery for fertility purposes.

131 Human Fertilisation and Embryology Authority, *supra* note 30, at para 8.3 and 8.14–15 (emphasis added).

132
*Id*. at para 8.15.

133 Ethics Committee of the American Society for Reproductive Medicine, *supra* note 47, at 944.

134
*Id*. at 944–945; 947 (emphasis added).

135 Wall and Testa, *supra* note 128, at 21.

136
*Id*, at 21.

137 Bruno and Arora, *supra* note 100, at W7.

138 Bruno and Arora, *supra* note 6, at 12.

139
*Id*. at 11.

140 Tonkens, *supra* note 116, at 10.

141 See Douglas, *supra* note 59, at 69–70.

142 Jackson, *supra* note 26, at 180.

143 For example, it has been suggested by Bayefsky and Berkman, *supra* note 82, at 356–357, that the fair innings argument and prudential lifespan account allow for the ranking of patients within the childbearing age group. It is further suggested by the same authors that women who are nearing the end of childbearing age should be prioritized over younger women of childbearing age to avoid them ‘aging out’.

144 Bruno and Arora, *supra* note 6.

145
*Id*. at 11–12; Bruno and Arora, *supra* note 100, at W7.

146
*Id*. at 12, emphasis added.

147
*Id*. at 12.

148
*Id*.

149
*Id*.

150 Noting that their proposal applies to both selection and allocation.

151 Section 1(1) of the Children Act 1989.

152 Note I would also be opposed to any proposals for welfare to occupy paramount status in any other child welfare assessments conducted as a prerequisite to fertility treatment.

153 Bayefsky and Berkman, *supra* note 82, at 359.

154 Bruno and Arora, *supra* note 100, at W7.

155 Note that it is possible that single women may be de-prioritized or rejected from UTx programs entirely due to lack of adequate social support given the significant physical burdens of treatment and the suggested importance of social support systems in post-transplant recovery—see Kelsey N. Berry et al., *Should Lack of Social Support Prevent Access to Organ Transplantation?*, 19 AM. J. BIOETH. 13 (2019). However, it should be recognized that this is not without controversy. For more detailed discussion on concerns about transparency and justice arising from the varying use of assessments of social support in determining suitability, see Berry et al. (above) and Keren Ladin et al., *How Important is Social Support in Determining Patients’ Suitability for Transplantation? Results from a National Survey of Transplant Clinicians*, 44 J. MED. ETHICS 666 (2018).

156 Horvat and Iltis, *supra* note 110, at 992.

157 NHS Blood and Transplant, *Assessment of Allocation Policies for Organs from Deceased Donors: Policy POL187/3*, 3 https://nhsbtdbe.blob.core.windows.net/umbraco-assets-corp/18118/pol187.pdf (accessed June 10, 2022) (emphasis added).

